# Functional Genomic and Advanced Genetic Studies Reveal Novel Insights into the Metabolism, Regulation, and Biology of *Haloferax volcanii*

**DOI:** 10.1155/2011/602408

**Published:** 2011-11-30

**Authors:** Jörg Soppa

**Affiliations:** Institute for Molecular Biosciences, Goethe University Frankfurt, Biocentre, Max-Von-Laue-Stra*β*e 9, 60438 Frankfurt, Germany

## Abstract

The genome sequence of *Haloferax volcanii* is available and several comparative genomic *in silico* studies were performed that yielded novel insight for example into protein export, RNA modifications, small non-coding RNAs, and ubiquitin-like Small Archaeal Modifier Proteins. The full range of functional genomic methods has been established and results from transcriptomic, proteomic and metabolomic studies are discussed. Notably, *Hfx. volcanii* is together with Halobacterium salinarum the only prokaryotic species for which a translatome analysis has been performed. The results revealed that the fraction of translationally-regulated genes in haloarchaea is as high as in eukaryotes. A highly efficient genetic system has been established that enables the application of libraries as well as the parallel generation of genomic deletion mutants. Facile mutant generation is complemented by the possibility to culture *Hfx. volcanii* in microtiter plates, allowing the phenotyping of mutant collections. Genetic approaches are currently used to study diverse biological questions–from replication to posttranslational modification—and selected results are discussed. Taken together, the wealth of functional genomic and genetic tools make *Hfx. volcanii* a bona fide archaeal model species, which has enabled the generation of important results in recent years and will most likely generate further breakthroughs in the future.

## 1. Introduction

On the one hand, biology tries to represent the biodiversity that is found in nature; in contrast to other natural sciences such as physics, this makes it nearly impossible to derive general rules. On the other hand, biology has profited tremendously from studies of so-called “model species,” for example, *Escherichia coli* and *Bacillus subtilis* for Gram-negative and gram-positive bacteria, *Saccharomyces cerevisiae *and *Schizosaccharomyces pombe* for fungi, *Drosophila melanogaster* for developmental biology, and, increasingly, the mouse for higher eukaryotes. Therefore, it is desirable to have a few model species for the third domain of life, the Archaea, and *Haloferax volcanii* has already been discussed as a suitable candidate [1]. There has been rapid progress in understanding the biology of *Hfx. volcanii in *the last five years. Therefore, results obtained in recent years will be reviewed here, with attention to (1) “functional genomics,” that is, the bioinformatic analysis of the genome sequence, transcriptomics, translatomics, proteomics, and metabolomics and (2) the genetic system of *Hfx. volcanii* and its application in characterizing a variety of central biological functions.

**Figure 1:**
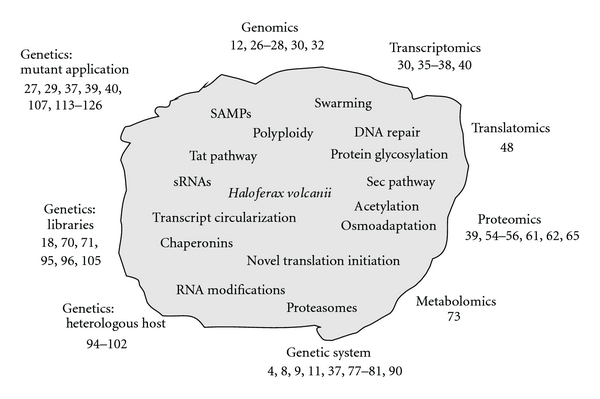
The figure schematically shows the pleiomorphic cell morphology of *H. volcanii. *Inside of the cell selected topics are listed that are discussed in this review. Outside of the cell the titles of selected chapters of this review are shown together with the respective most important publications (compare reference list).

*Hfx. volcanii* was isolated from the Dead Sea 35 years ago and named in honor of Benjamin Elazari Volcani [2], who was the first to determine that the Dead Sea, in spite of its name, contains many microorganisms [3]. *Hfx. volcanii* is pleomorphic, has an extremely high range of salt tolerance, and is able to grow in environments from 0.7 M M to 5 M NaCl, with an optimum concentration around 2.2 M [4]. It has a versatile metabolism and can be grown in simple synthetic media with a variety of sugars, amino acids, glycerol, pyruvate, or acetate as the sole carbon and energy source. In addition to aerobic growth, it can also grow anaerobically using nitrate, DMSO, or TMAO as an electron acceptor. Under optimal conditions, it has a doubling time of around three hours [5], and even faster growth has been reported [6]. Under certain conditions, it is motile [7], and it can form biofilms on several surfaces (Vogel and Soppa, unpublished data). A transformation system was established more than 20 years ago [8, 9], and many useful features have been added since then (recent examples are given below). *Hfx. volcanii* has a natural gene transfer system [10, 11] that can be applied to combine mutations and thus speed up molecular genetics. The genome has been sequenced, and, as will be described below, all methods of functional genomics have been established. *Hfx. volcanii* has been used to study many central biological processes, including replication, DNA repair, transcription and transcriptional regulation, translation, protein export, posttranslational modification of proteins, protein degradation, and various aspects of metabolism. Selected examples from recent years will be described below. Figure 1 gives an overview of the different topics and respective important references.

## 2. Genomics

The availability of the genome sequence of a species under investigation is a prerequisite for bioinformatic analyses and many experimental approaches. An initial *Hfx. volcanii* genome project was started more than 10 years ago but ultimately failed, and further projects were held back for years. Subsequently, TIGR began a second genome project and generated a draft version of the genome sequence by 2005. The genome sequence was published in 2010 [12], but the draft version was available to the *Hfx. volcanii* community years before and enabled the establishment of proteomics and bioinformatic genome analyses, among other fields (see below). The annotation of the *Hfx. volcanii* genome made use of the reference genome of *Hbc. salinarum* R1, whose annotation is of extremely high quality because it is based on extensive experimental proteomics [13]. Therefore, small proteins that had been experimentally found in a study characterizing the “low molecular weight proteome” of *Hbc. salinarum* are annotated in *Hfx. volcanii* [14]. Furthermore, several additional genomes of haloarchaea have recently been sequenced, enabling the application of comparative genomics to enhance the quality of annotation. The genome sequence and many additional features can be found at the “archaeal genome browser” and “halolex” websites (http://archaea.ucsc.edu/; http://www.halolex.mpg.de/) [15, 16].

The genome sequence underscored the high quality of earlier mapping results [17]. It was determined that four of the five replicons contain Cdc6-associated replication origins [18]. Therefore, *Hfx. volcanii* is now described as containing one major chromosome of 2.8 Mbp, three smaller chromosomes of 636 kbp, 438 kbp, and 85 kbp (pHV 4, 3, 1) and one plasmid of 6.4 kbp (pHV2) [12]. The GC content of the genome is rather high at 65%, but this is similar to other haloarchaea, with the exception of *Haloquadratum walsbyi*. The great difference in GC content is in the coding regions (65%), in contrast to the non-coding regions (58%), and the high number of insertion (IS) IS elements in haloarchaea has led to the theory that the high GC content is caused by evolutionary selection away from the AT preference of the IS elements. This difference in GC content would minimize the frequencies of insertion into additional places on the genomes of haloarchaea and would be especially important for coding regions [12]. The theory also holds true for *Hqr. walsbyi*, a species that has a very low GC content of 48% and uses the opposite technique to avoid the ideal GC content for IS element insertion, that is, by decreasing the GC content.

The major chromosome has two origins of replication [18]. Thus, *Hfx. volcanii* is added to the growing list of archaeal species with more than one replication origin, a characteristic that was first discovered for *Sulfolobus solfataricus* [19, 20]. *Hfx. volcanii* was found to be highly polyploid, and it harbors approximately 15 genome copies during exponential growth and 10 during the stationary phase [21]. This is similar to *Hbc. salinarum*, which has an even higher genome copy number. Also, several additional species of Euryarchaeota are oligoploid or polyploid, while all characterized species of Crenarchaeota have been found to be monoploid [22]. The low rate and unusual pattern of spontaneous mutations is most likely due to the polyploidy of *Hfx. volcanii* [23].

In total, the genome of *Hfx. volcanii* encodes 4 063 protein-encoding genes. Like all other haloarchaea and the halophilic bacterium *Salinibacter ruber, Hfx. volcanii* also uses the “salt-in” strategy of osmotic adaptation, that is, the salt concentration in the cytoplasm is as high as in the environment. As a result, all cellular constituents are evolutionarily adapted to function under high salt conditions. Therefore, the proteomes of all these halophiles exhibit the same unique features separating them from all other organisms. The proteins are enriched in aspartic and glutamic acid, valine, and threonine, and, in contrast, the fractions of lysine, methionine, leucine, isoleucine, and cysteine are decreased [24]. In addition, halophilic proteins show a lower hydrophobicity (or higher hydrophilicity) than proteins of other organisms [24].

The genome sequence revealed that *Hfx. volcanii* is characterized by an expansion of the ABC transporter family but not other families of transporters. In total, 69 predicted gene clusters for ABC transporters were found [12], underscoring the versatile metabolism of *Hfx. volcanii* noted earlier. In accordance with this versatile metabolism, the *Hfx. volcanii* genome encodes a large number of signal transduction components, including 135 one-component systems, that is, proteins that contain both a sensory and a regulatory domain [12]. 

Another peculiarity of haloarchaea is the extensive use of the twin arginine translocation (TAT) pathway of protein secretion [25]. It has been proposed that this feature is also an adaptation to the high salt environment and allows for the chaperone-assisted folding of proteins in the cytoplasm before they are secreted. The high salt environment might inhibit spontaneous folding of proteins exported in an unfolded form via the sec pathway. The program “TATFIND” has been introduced for the genomewide prediction of proteins that are putatively exported via the TAT pathway [26]. Recently, it was complemented by the program “TatLipo” because it was noted that, in *Hfx. volcanii* and other haloarchaea, many TAT pathway substrates contain a lipobox and are presumably covalently anchored to a lipid of the cytoplasmic membrane [27]. Comparative genomics revealed that, in six species of haloarchaea, the fraction of predicted lipoproteins among TAT substrates is 50% or higher, while this fraction is around 0% in 20 additional archaeal and bacterial genomes [27]. 

The initial work on protein export in *Hfx. volcanii* led to a change in the concept of protein transport in bacteria as well. Based on studies with *E. coli*, it had long been thought that TAT pathway substrates are very rare. TAT substrates were believed to be limited to a few periplasmic proteins containing FeS clusters that must be incorporated into the protein by chaperones in the cytoplasm. However, work in predicting TAT substrates in all genomes known at that time revealed that several bacterial species have more than 90 TAT pathway substrates (*Streptomyces coelicolor, Mezorhizobium loti, Sinorhizobium meliloti)*, while other bacterial species apparently do not use the pathway at all (e.g., *Borrelia burgdorferi, Ureaplasma urealyticum, Streptococcus pneumoniae*) [26].

The genome sequence of *Hfx. volcanii* was also used for a bioinformatic prediction of the modification sites of stable RNAs, the guide RNAs involved in RNA modification, and RNA-modifying enzymes [28, 29]. The number of predicted modifications was astonishingly low, and this was also interpreted as an adaptation to the high salt cytoplasm because RNA stability is higher under high salt conditions. It was hypothesized that the modifications present in *Hfx. volcanii* are particularly important for the mechanism of translation and therefore cannot be replaced through evolution. If this turns out to be true, *H. volcanii* would be a model for a minimal set of essential RNA modifications and could be used to study their biological functions.

A further application of the *Hfx. volcanii* genome sequence was the genome-wide bioinformatic prediction of conserved motifs in intergenic regions using comparative genomics. Conserved motifs in intergenic regions include small non-coding regulatory RNAs (sRNAs) and presumed regulatory elements in 5′- and 3′-UTRs. Experimental validation revealed that more than half of the predicted sRNAs are expressed under standard conditions and enabled the characterization of differential expression [30]. 

Recently, it was discovered that proteins in *Hfx. volcanii* are modified by covalent attachment of “SAMPs”, Small Archaeal Modifier Proteins. SAMPs are ubiquitin-like (Ubl) proteins that become covalently attached to other proteins, a modification that marks them for degradation via the proteasome [31]. This outstanding discovery solved the puzzle that archaea contain a proteasome, which is homologous to the eukaryotic proteasome, but not ubiquitin. Ubiquitin marks eukaryotic proteins for degradation via the proteasome, and the lack of a ubiquitination system inhibited understanding of the onset of protein degradation in archaea. It was shown that SAMPs can be covalently linked to the epsilon amino group of lysines via a glycine of the highly conserved double glycine motif at the C-terminus. “SAMPylated” protein conjugates were isolated by immunoprecipitation, and 34 proteins were identified, including metabolic enzymes, stress response proteins, and DNA- and RNA-binding proteins. It was also revealed that SAMPs can attach to themselves, meaning that poly-SAMPylation occurs, analogous to the polyubiquitination seen in eukaryotes (compare Section 10 for results on chaperonins and protein folding).

The discovery of SAMPs triggered a comparative genomic study of the whole Ubl protein family in archaea [32]. It was revealed that most archaea contain members of two major groups of Ubl proteins, the ThiS family and the MoaD family. The gene context analysis of *ubl* genes in archaeal genomes led to the prediction that the primary function of the MaoD family members is indeed Mo/W cofactor biosynthesis, as the name implies. However, an important finding was that ThiS family members are often associated with highly conserved and, most likely, essential genes encoding proteins related to translation, especially tRNA modification. It was proposed that the ancestral function of Ubl proteins was the biosynthesis of modified nucleotides in tRNAs, which is important for translation fidelity. According to this hypothesis, Ubl proteins would have acquired additional functions only later, for example, MoCo, thiamine biosynthesis, and protein modification. In this view, Ubl proteins today act as general devices of protein quality control on several levels, including translational fidelity and protein degradation. 

The existence of a multifunctional Ubl protein in *H. volcanii* was experimentally verified [33]. Inactivation of a gene encoding a protein named UbaA (Ubl activating enzyme of archaea, HVO_0558) resulted in the loss of protein SAMPylation by SAMP1 and SAMP2. It was also shown that *H. volcanii* is unable to grow via DMSO respiration in the absence of UbaA, and that the mutant does not contain DMSO reductase activity, in contrast to the wild type. It was proposed that SAMP1 and UbaA are involved in sulfur transfer to an intermediate of MoCo biosynthesis. A third function of UbaA is the thiolation of tRNA^Lys^_UUU_ and, likely, additional tRNAs. It was proposed that the heat-sensitive phenotype of the *ubaA*-deletion mutant is caused by the lack of tRNA thiolation [33], as this had been earlier found to be true for *Thermus thermophilus* [34]. These excellent results nicely verified the predictions based on comparative genome analysis.

## 3. Transcriptomics

The genomewide parallel quantification of all transcript levels can today be routinely performed and has been proven to be extremely powerful. DNA microarray technology was introduced more than 10 years ago, and recently, alternative techniques such as next-generation sequencing of cDNA libraries have been established. DNA microarray analysis of the *Hfx. volcanii* transcriptome was introduced in 2003, several years before the genome sequence became available. Therefore, an alternative approach to the typical genome sequence-based DNA microarray technology was used. A genomic library composed of plasmids with genomic fragments with an average length of 1.5 kbp was constructed and converted into a PCR product library. This library was used to generate a shotgun DNA microarray with a onefold coverage of the genome [35]. Accordingly, the probability that a specific gene is represented on the DNA microarray is 67%. However, the aim of transcriptome studies is to yield an overview of the regulation of biological pathways and processes, and therefore, it is not necessary that every single gene be represented. The probability that none of the components of a whole pathway or even of a protein complex, such as an ABC transporter, are represented is extremely low. For example, the probability that none of the 11 genes encoding the eight TCA cycle enzymes are represented on the DNA microarray is 5 × 10^−6^. The 3000 genomic fragments comprising the DNA microarray were sequenced, and the genes represented by the genomic fragments were identified. 

The first application was the characterization of kinetics of transcriptome changes following a switch from casamino acids to glucose as the sole carbon and energy source [35]. The expected upregulation of the glucose degradation pathway was revealed. Many processes were downregulated, including gluconeogenesis, the citric acid cycle, electron transport, ATP synthesis, ribosome biosynthesis, and replication. This is in line with the fact that *Hfx. volcanii* “prefers” casamino acids; the growth rate, ribosome content, and central metabolism are down-regulated after the switch to glucose. Transcriptome studies are discovery-driven approaches and, as such, typically yield unexpected results. In this example, unexpected results were obtained related to acetate metabolism, methyl-malonyl-CoA metabolism, ABC transporters, and protein phosphorylation [35]. Notably, two classes of kinetically coregulated genes were only transiently induced shortly after the shift from casamino acids to glucose and could not have been identified by the most commonly used comparison of two steady states [35]. Most of the encoded proteins have no known function, indicating that the current knowledge about the molecular details of transition processes is very limited.

The DNA microarray was also used to identify genes induced during growth on xylose as the sole carbon source and thereby enabled the elucidation of the xylose degradation pathway of *Hfx. volcanii *[36]. The enzymatic reactions catalyzed by the encoded enzymes were biochemically analyzed. In addition, several mutants were generated that proved that the respective genes are essential for xylose degradation in *Hfx. volcanii* and that no redundant pathway exists [36].

Another application of the DNA microarray was the identification of a gene encoding a tryptophanase that has a nearly hundredfold increased transcript level after the addition of tryptophan, while the transcript levels of all other genes remained unchanged [37]. The *tna* promoter was also shown to be inducible after it was transferred to various plasmids, and it is currently being used by quite a number of groups for conditional protein production or conditional gene silencing (a few examples are mentioned below).

In several cases, the DNA microarray has been used to compare the transcriptomes of deletion mutants and the isogenic wild type [38, 39] (Dambeck and Soppa, unpublished data). In several cases, this enabled the unraveling of the biological roles of proteins or of small non-coding RNAs (sRNAs). For example, in one mutant, a DNA microarray analysis revealed a change in the transcript levels of genes involved in chemotaxis or encoding components of the flagellar apparatus, and a subsequent analysis on swarm plates verified a different swarming velocity of the mutant compared to the wild type [unpublished results]. 

It should be noted that, based on the open platform that was chosen, it is easy to add further probes, which is impossible for several other microarray formats. For example, probes for sRNAs were added, and the microarray can now be applied to analyze the differential expression of sRNA genes and protein-encoding genes of *Hfx. volcanii* simultaneously. As mentioned above, the DNA microarray has been used to analyze whether bioinformatically predicted small RNAs are expressed and thus can be proven to be real genes [30]. It was also used to identify sRNAs that were bound to and could be copurified with the haloarchaeal Lsm protein. These results proved that the haloarchaeal Lsm protein is involved in the function of many small RNAs in a similar manner to its homolog in bacteria, the Hfq protein of *E. coli* [40]. In summary, the DNA microarray based on a random genomic library has proven to be a valuable tool for studying various biological processes, including the differential regulation of a single gene or of several hundred genes.

Recently, a second microarray platform was established, a whole-genome Nimblegene DNA microarray (Thane Papke, personal communication; robertson.papke@uconn.edu). This microarray is currently being used by two groups of the community, for example, to elucidate the components of the DNA import system of *Hfx. volcanii*. Therefore, usage of DNA microarray technology is now spreading in the *Hfx. volcanii* community, and important discoveries can be expected in the near future using both microarray platforms.

## 4. Translatomics

A DNA microarray is not only used for the analysis of transcript levels but can also be applied for the characterization of translational efficiencies of all transcripts present under specific conditions. The experimental design of a translatome analysis is considerably more complex than that of a transcriptome analysis and typically includes the following steps: (1) the preparation of a cytoplasmic extract under mild conditions that preserve the complexes of transcripts and translating ribosomes (polysomes), (2) the separation of free transcripts that were not translated at the time of cell disruption from polysomes using density gradient centrifugation (ribosomal profiling), (3) the isolation of RNA from both fractions, (4) the comparison of both fractions using DNA microarrays or second generation sequencing, and (5) the identification of transcripts with nonaverage translation efficiencies. For the identification of differential translational control, at least two different conditions have to be compared. 

About 20 such studies have been performed with eukaryotes, that is, *Saccharomyces cerevisiae, Arabidopsis thaliana,* and human cell lines. All of these studies identified translationally regulated genes, whose fractions varied from about 1% to 25%, depending on the species and the applied conditions [41–47]. Until now, no translatome analysis has been reported for any bacterial species. However, one study has been performed with two species of halophilic archaea, *Haloferax volcanii* and *Halobacterium salinarum* [48]. The translatomes of exponentially growing and stationary phase cultures were compared, and it was revealed that 10% and 20%, respectively, of all transcripts were under differential growth phase-dependent translational control. Thus, the fraction of translationally regulated genes in haloarchaea is as high as in eukaryotes, and translational regulation seemingly evolved prior to the development of eukaryotes. It will be interesting to unravel whether bacteria also use translational control for the regulation of gene expression and whether the fraction of regulated genes is equally high. 

The high fraction of translationally regulated genes in haloarchaea produced studies aiming to unravel the molecular details of the regulatory mechanism. It was revealed that 2/3 of the transcripts of *Hfx. volcanii* are leaderless and that Shine-Dalgarno (SD) motifs are very scarce. Surprisingly, it was found that translation is initiated by a “novel” mechanism for about 1/3 of all transcripts that are characterized by a 5′-UTR lacking a SD motif [49, 50].

In addition, it was shown that 5′- and 3′-UTRs together are sufficient to transfer translational regulation from the native transcript to a reporter transcript and that the direction of translational regulation is encoded in the 3′-UTR [51]. Currently, the molecular details of 3′-UTR's influence on translational efficiency at the 5′ end of transcripts are being studied. In eukaryotes, transcript circularization is thought to be a general phenomenon. It is mediated by a common interaction of the cap-binding translation initiation factor eIF4E and the polyA-binding protein Pabp with the initiation factor eIF4G [52, 53]. However, in haloarchaea, the mechanism must be different because they have mRNAs lacking polyadenylation and are devoid of orthologs of all three eukaryotic proteins.

## 5. Proteomics

The availability of the draft genome sequence to the community several years prior to the final genome sequence publication enabled the early establishment of bona fide modern proteomics. First, the methods for sample preparation and 2D gel electrophoresis were optimized to enable a reproducible representation of the cytoplasmic proteome of *Hfx. volcanii* [54, 55]. Subsequently, the adaptation of the *Hfx. volcanii* to different salt concentrations was analyzed [56]. Cultures were grown at the optimal NaCl concentration of 2.1 M and at an enhanced concentration of 3.5 M, and the steady state cytoplasmic proteomes were compared by 2D gel electrophoresis. Forty-four proteins with differential concentrations were detected, and 18 of these were identified by peptide mass fingerprinting. At the optimal salt concentration of 2.1 M NaCl, 15 proteins had a higher concentration or were uniquely detected, five of them ribosomal proteins. This is in line with an earlier observation that the ribosome content of *Hfx. volcanii* is sensitive to the growth rate [35], as had been described before for *E. coli* and other bacteria [57]. However, three proteins of *Hfx. volcanii* were up-regulated under the suboptimal high salt concentration of 3.5 M NaCl. Among them was a homolog of the *E. coli* “phage shock protein,” PspA, which is a bacterial transcription factor that is upregulated in response to a wide variety of stress conditions [56]. A similar experiment has also been performed with the extreme halophile *Hbc. salinarum*, which was grown at the optimal concentration of 4.3 M NaCl as well as at suboptimal concentrations of 2.6 M and 5.1 M NaCl [58]. In this case, comparison of the cytoplasmic proteins of cells grown under optimal and high salt conditions resulted in the identification of 50 proteins with differential concentrations. Differential regulation was more evenly distributed, as 30 proteins were upregulated under optimal conditions (the highest fraction were again ribosomal proteins), while 20 proteins were upregulated under high salt concentrations (the highest fraction were proteins of unknown function). Therefore, it appears that the mechanism of adaptation to nonoptimal salt concentrations is not conserved in different species of haloarchaea.

Several proteomic studies concentrated on the characterization of protein degradation through the proteasome. The proteasome is composed of four stacked, heptameric rings of subunits, and its overall structure is highly conserved in archaea and eukaryotes. While the heptameric rings of the eukaryotic proteasome are composed of seven different subunits, the composition of the archaeal proteasome is less complex. *Hfx. volcanii* has one beta subunit that forms homoheptameric rings and two alpha subunits that are differentially expressed in the exponential phase and the stationary phase, and thus, it is capable of forming at least two different types of proteasomes [59, 60]. To clarify the biological role of the proteasome, cells were incubated with the proteasome-specific inhibitor clasto-lactacystin-beta-lactone, and the proteomes of treated and untreated cultures were compared [61]. After proteasome inhibition, the number of spots visible on 2D gels was much higher compared to control cultures; that is, the inhibition of proteasome function led to an increase from 627 to 1036 spots, indicating that proteasomes are involved in degrading a large variety of different proteins in haloarchaea [61]. The identities of about 20 proteins were determined. Among them were five ribosomal proteins as well as metabolic enzymes and cell division proteins, underscoring the general role of the proteasome for protein turnover in haloarchaea [61]. In an additional approach to identify proteasome substrates, the proteasome-activating nucleotidase PanA, which unfolds proteins prior to their degradation by the proteasome, was inactivated by the deletion of its gene [62]. The *panA* mutant had a longer doubling time and a reduced growth yield compared to the isogenic wild type, underscoring the importance of protein turnover for *Hfx. volcanii*. Comparison of the phosphoproteomes using the stain Pro-Q diamond led to the detection of twice as many spots in the mutant compared to the wild type (64 compared to 31 spots). Using three different approaches, phosphoproteins were enriched from the cytoplasmic extracts (IMAC; MOAC; IMAC + MOAC), and the proteins of the enriched mixture were identified by peptide mass fingerprinting after separation by reversed-phase HPLC. In total, 625 proteins were identified, 98 of which were unique to the mutant. The 98 mutant-specific proteins contained proteins of many different functional classes, for example, metabolic enzymes, cell-cycle-related proteins and DNA binding proteins. One notable difference was the enrichment of a variety of proteins of the phosphate regulon, indicating that the loss of PanA led to cell stress, in accordance with the lower growth rate [62]. The identification of the two SAMP proteins of *H. volcanii* that via covalent attachment mark proteins for degradation through the proteasome has already been described above (in section “Genomics”). The widespread distribution of genes for SAMPs in archaeal genomes indicates that conjugation of proteins with SAMPs is a very old and general mechanism in archaea to mark proteins for degradation via the proteasome. Thus, the combination of proteomics with *H. volcanii* and comparative genomics enabled the discovery of a novel and yet widespread molecular mechanism for protein turnover.

Another approach was aimed at identifying the protein inventory of the *Hfx. volcanii* cytoplasm [63]. In addition to the classical 2D gel electrophoresis-based approach, five additional approaches were applied, leading to the total identification of 1 296 proteins, representing 32% of the theoretical cytoplasmic proteome. The N-termini of 236 proteins could be analyzed, and it was revealed, that in 70% of these proteins, the N-terminal methionine was removed and/or the initiator methionine or the penultimate amino acid (after methionine removal, the N-terminal amino acid) was acetylated at the alpha amino group. As expected, none of the N-termini were formulated, in contrast to the N-termini of bacterial proteins. In summary, analyses of the *Hfx. volcanii* cytoplasmic proteome have been very informative about the protein inventory, N-termini, posttranslational modifications, differentially regulated protein levels and have notably yielded spectacular results concerning protein turnover. Proteome analysis of *Hfx. volcanii* has as yet been limited to the cytoplasmic proteome. However, additional benefits can be made by comparison with the results of proteome studies with *Hbc. salinarum* that characterized the membrane proteome or the low-molecular-weight proteome [14, 64, 65].

## 6. Metabolomics

During recent years, metabolomics has made rapid progress and matured from the quantification of only very few metabolites to the bona fide parallel quantification of hundreds of metabolites [66]. Due to the very different chemical behavior of different metabolites, metabolomics is still demanding. Currently, there is only a single study with *Hfx. volcanii* involving a metabolomic approach. The genome of *Hfx. volcanii* harbors three gene clusters that encode subunits of three 2-oxoacid dehydrogenase complexes, but the substrates and biological roles for all three enzyme complexes remained obscure for a long time [67–72]. In an attempt to apply genetics to solve the question, seven strains were constructed that contained the three possible single gene deletions, the three possible double deletions, and a triple gene deletion [73]. Comparison of the extracellular metabolomes of wild-type and mutants revealed that the wild type depleted complex media of isoleucine, while mutants lacking one of the 2-oxoacid dehydrogenase complexes (OADHC-1) were unable to do so. Subsequent analyses verified that OADHC-1 is indeed essential for degrading isoleucine but not the other branched-chain amino acids [73].

## 7. Genetics: From a Transformation System to Genome Design and Phenotyping of Mutant Collections

*Hfx. volcanii* and *Hbc. salinarum* (at that time named *Hbc. halobium*) were the first and, for many years, the only archaeal species for which a transformation system was available [8, 9]. Over the years, more and more molecular genetic tools were added, and today, several compatible plasmids, several reporter genes, a regulated promoter, and advanced cloning strategies have been established. The most commonly used reporter genes are the *bgaH* gene from *Hfx. alicantei* encoding a salt-adapted beta-galactosidase [74], the *dhfr* gene from *Hfx. volcanii* encoding Dihydrofolate reductase [75], and a salt-tolerant variant of the green fluorescent protein [76]. 

An important step was the development of an approach for the generation of chromosomal deletion mutants [77]. Subsequently, several auxotrophic strains and plasmids carrying the respective genes were generated, which added additional selection schemes and facilitated the generation of in-frame deletion mutants [78]. Optimized cloning procedures have been reported [79–81], two of which are so robust that vector generation in microtiter plates is possible [79, 81]. These approaches make the generation of a library of mutants that contain single deletion mutants of all genes of *Hfx. volcanii* technically feasible. This approach has been performed with several model species of bacteria, and it has been proven to be extremely powerful for the elucidation of gene function [82–84]. The approach of genome-wide mutant production and their phenotypic comparison with the isogenic wild type can only be successful when parallel cultivation of mutants under many different conditions is feasible. To this end, two different conditions for the cultivation of *Hfx. volcanii* in microtiter plates have very recently been reported. One approach made use of an automatic system and thus has the advantage of minimal handling efforts, but it involves lengthy generation times and low growth yields, making the quantification of subtle differences between mutants and wild type challenging [79]. The other approach depends on individual growth measurements, requiring hands-on time of researchers, but enables rather fast growth with rates that are not much lower than growth in Erlenmeyer flasks with optimal aeration [4].

As mentioned above, haloarchaea were the first group of archaea for which genetic techniques were established, and they were long at the leading edge of development of molecular genetic techniques. However, in recent years, tremendous progress has been made with other model species of various archaeal groups, the progress of which has been reviewed recently [85]. The magnitude of progress can be estimated when the current possibilities are compared to the state of the art only five years earlier [86, 87]. Even a gene transfer system for the hyperthermophilc *Pyrococcus furiosus* has been reported [88, 89]. Therefore, several archaeal model species now offer an increasingly inclusive set of genetic, biochemical, microbiological, cell biological and structural biological tools. While *Hfx. volcanii* was leading in molecular genetics, it has had weaknesses in other areas; for example, proteins produced heterologously in *E. coli* were unfolded, and subsequent refolding had to be optimized for each protein individually and was not always successful. Recently, the first genuine haloarchaeal expression vector was constructed that enables the conditional over-production of his-tagged fusion proteins that can easily be isolated by affinity purification [90]. This approach holds the promise that proteins will be natively folded after homologous production and can be applied for enzyme kinetic characterizations as well as for crystallization and structural studies.

## 8. Genetics: Application of *Hfx. volcanii* as a “Natural Null Mutant”

Studying a biological process not in its native host but in a heterologous host instead has often proven to be extremely powerful [91–93]. Advantages include the ability to detect novel genes encoding specific functions, the ease in replacing wild-type genes with mutated versions, and the certainty that the studied genes are not only required but also sufficient for the process under investigation. Several projects used *H. volcanii* as a heterologous host to characterize functions not encoded in its genome or to discover novel genes. Fifteen years ago, the *arcC* gene of *Hbc. salinarum* was expressed in *Hfx. volcanii*, and it was verified that it codes for a carbamate kinase, one of the four proteins of the arginine fermentation pathway [94]. In an elegant approach, a deletion mutant of the dihydrofolate reductase gene was used to identify nonorthologous genes from the genomes of *Hbc. salinarum* and *Haloarcula marismortui* that encode enzymes for an alternative pathway for reduced folate biosynthesis. Prior to the study, these genes had no known function [95, 96]. Recently, the biological function of a gene annotated as *fabG1*, which is one of six *fabG* paralogs of *Haloarcula hispanica*, was clarified [97]. It was shown that *fabG1* encodes a polyhydroxyalkanoate-(PHA-) specific acetoacetyl coenzyme A reductase. Heterologous expression of *fabG1*, but not of the other five *fabG* paralogs, together with the *phaEC* genes eoncoding PHA synthase, reconstructed the PHA synthesis pathway of *H. hispanica* in *Hfx. volcanii*.

*Hfx. volcanii* was used extensively to characterize the three gas vesicle gene clusters of *Hbc. salinarum* and *Haloferax mediterranei*, resulting in more than 20 publications. For example, the minimal number of genes for gas vesicle formation was determined, and the role of the minor structural protein GvpC was clarified. In addition, the regulatory roles of GvpD and GvpE were characterized, the requirements for their interaction were elucidated, and the interaction of GvpE with TATA box-binding proteins *in vivo* was studied. Furthermore, the regulation of gas vesicle synthesis was studied (e.g., [98–102], and references therein).

A different angle for the heterologous production of proteins in *Hfx. volcanii* is metabolic engineering, which ultimately aims to use the species for biotechnological applications. A first example is the production of the pyruvate decarboxylase of *Zymomonas mobilis* in *Hfx. volcanii*, with the putative future goal of bioethanol production [103]. A recent review summarizes current and potential future biotechnological applications of halophilic microorganisms [104].

## 9. Genetics: Application of Plasmid Libraries

The high transformation efficiency of 10^6^ transformants per microgram of DNA enables the application of libraries. One example was the selection of plasmids containing active promoters *in vivo* from a random library of more than 10^6^ clones [105]. Fourteen positions upstream of a reporter gene were randomized, and the reporter enzyme activity used for selection. Thus, this represented the *de novo* construction of active promoter elements from a random pool of sequences. In addition, plasmids with low, intermediate, and high promoter strength were generated, which were later used to tune the expression of genes to the desired level.

A second example was the complementation of nitrate respiration-deficient mutants of *Hfx. volcanii* with a genomic library of the wild-type genome. Using this approach, eleven genomic regions with essential genes for nitrate respiration could be identified, including genes for ABC transporters, enzymes for glucose degradation, a 2-oxoacid dehydrogenase complex, a transcriptional regulator, and proteins with unknown function [70, 71] (Wanner and Soppa, unpublished data).

As already mentioned above, plasmids libraries were also applied to identify replication origins of *Hfx. volcanii* [18] and to use *Hfx. volcanii* to unravel reduced folate synthesis of *Hbc. salinarum* [95]. All four studies exemplify the ease by which genetic strategies originally developed for *E. coli* can be adapted to *Hfx. volcanii*, so that the whole power of genetic technologies can be applied.

## 10. Genetics: Application of Mutants to Characterize Important Biological Processes

The so-called Pop-In-Pop-Out method for the construction of *Hfx. volcanii* mutants was introduced in 2003, and in 2004, further strains and plasmids were added [77, 78]. The method rapidly spread around the world, and until 2008, the generation of more than 20 mutants had been published by various groups (an overview is given in Table 2 of [106]). Since then, the ease of construction has led to the generation of many mutants, and the number must currently be well over 100. As a tool for the community to gain an overview of the mutated genes and inhibit multiple generation of the same mutant by different groups, a mutant database is currently under construction (Friedhelm Pfeiffer, personal communication). It will be accessible to the community via the *Hfx. volcanii* website (http://www.haloferax.org/). The mutants are used to study a variety of biological functions and pathways, and a few examples will be shortly discussed, from DNA biochemistry to posttranslational processes.

In a genetic approach to study replication, a variety of mutants were constructed, with an emphasis on genes encoding proteins that travel with the replication fork [107]. Another aim is to understand the different DNA repair pathways of *Hfx. volcanii* and unravel their relative importance under various conditions [108–111]. One unexpected result was that homologous recombination, which was thought to be the main mechanism for the repair of double-strand breaks in prokaryotes, is repressed by Mre11-Rad50 in *H. volcanii*. It was hypothesized that, in a polyploid species in which multiple double-strand breaks of different chromosomes might arise, repair by homologous recombination might lead to strange DNA molecules that are disadvantageous for the cell. Consequently, it was predicted that homologous recombination as a means for double-strand break repair might also be repressed in other polyploid prokaryotes. Notably, a genetic assay was established that allows the quantification of the usage of different repair pathways in *H. volcanii in vivo*.

In a bioinformatic approach, all RNA modifications have been proposed, and the enzymes catalyzing the formation of these modifications have been predicted [28]. Starting from this overview, the construction of approximately 30 mutants of genes putatively involved in RNA metabolism was attempted [79, 112]. Ten mutants could not be generated, and the genes are possibly essential. However, more than 20 mutants were successfully constructed and enabled the verification of predicted enzymatic functions in RNA biochemistry [79, 112]. 

Small non-coding regulatory RNAs (sRNAs) have been detected in all three domains of life. RNomics, bioinformatic predictions coupled to experimental validation and high-throughput sequencing have led to the identification of more than 200 sRNAs in *Hfx. volcanii* ([30, 39, 113], unpublished results). About 30 deletion mutants of sRNA genes have been constructed, and currently, phenotyping is applied to unravel biological functions of sRNAs in *Hfx. volcanii* [4] (unpublished data). A deletion mutant of the gene encoding the only Lsm protein in *Hfx. volcanii* had a severe growth defect and a pleiotropic phenotype, indicating that the Lsm protein is necessary for the function of many sRNAs [40]. 

Chaperonins are ubiquitous protein folding machines that are composed of two rings and have a “double doughnut” structure (compare section “Genomics” for protein degradation). *Hfx. volcanii* has three genes for group II chaperonins, and it was interesting to clarify whether all of them are essential, similar to the eight eukaryotic group II chaperonins. A mutant analysis revealed that all three single mutants can be generated, and thus, the three proteins are individually dispensable [114]. However, only two of the three possible double mutants could be constructed, and the simultaneous deletion of all three genes was impossible. These results clearly showed that the presence of at least one of the chaperonins is absolutely essential. The chaperonin genes were used to exemplify conditional silencing of essential genes in *Hfx. volcanii*. A tryptophan inducible promoter that enabled gene silencing and conditional protein depletion upon tryptophan removal was used [37].

As already described above, deletion mutants were also applied to characterize protein degradation via the proteasome. In addition, a variety of mutants were used to analyze protein secretion through the sec pathway, which exports proteins in an unfolded form, and the Tat pathway, which exports natively folded proteins (e.g., [27, 115–119]).

Genetic approaches were also used to study posttranslational reversible protein acetylation. Attempts to generate single and double mutants of genes for protein acetylases and deacetylases revealed that one of two protein deacetylases is essential. Furthermore, single-deletion mutants of all three acetylase genes could be constructed, but only two of the three possible double mutants could be obtained. Taken together, the results revealed that reversible protein acetylation is essential for *Hfx. volcanii* [120]. 

The combination of extensive genetic with biochemical analysis enabled the unraveling of the protein glycosylation pathway ([121–126] and references therein). In addition to the generation of mutants to verify the enzymatic activity, site-directed mutant versions were used for complementation and enabled the identification of residues important for catalysis. Last but not least, this project is now entering the realm of synthetic biology by adding genes from other haloarchaeal species, with the aim of transforming *Hfx. volcanii* into a “glycol engineering workshop.”

## 11. Outlook

Rapid developments in recent years have transformed *Hfx. volcanii* into a bona fide archaeal model species, with a sequenced genome and functional genomic, advanced genetic, biochemical, microbiological, and cell biological methods in place. Noteworthy, *Hfx. volcanii* and *Hbc. salinarum* are the only prokaryotic species with which translatome analyses have been performed, revealing that translational regulation in haloarchaea is as important as in eukaryotes. The existence of several chromosomes and the polyploidy of the major chromosome make *Hfx. volcanii* an attractive model in studying the evolution of parallel replicons and the evolutionary advantage of polyploidy. In addition, the pioneering results of discovering a novel mechanism for translation initiation and the novel family of SAMP proteins, which mark archaeal proteins for degradation, will produce further research that will yield results for the domain of archaea or even for all prokaryotes. 

Developments in the near future should include the establishment of more “focused proteomics” that center on the characterization of exported proteins, low-molecular-weight proteins or proteins with specific posttranslational modifications. In addition, the combination of different functional genomic methods with bioinformatics and modeling remains missing for *H. volcanii*, techniques that have been proven to be extremely powerful for *H. salinarum* (compare relevant publications of Nitin Baliga and Dieter Oesterhelt, e.g., [127, 128]). 

Furthermore, methods to study protein-protein interactions should be developed, for example, several Gfp variants that can be used to study protein interaction *in vivo* using FRET, introduction of a “haloarchaeal two-hybrid system” or the generation of more efficient tags for affinity isolation of protein complexes. Only a few structures of haloarchaeal proteins have been solved until now, in spite of the fact that it has been predicted that conditions for crystallization of various haloarchaeal proteins should be more similar than for the crystallization of nonhaloarchaeal proteins. It has been argued that due to the adaptation to high cytoplasmic salt concentrations the physicochemical characteristics of haloarchaeal proteins would be more similar than those of proteins of low salt organisms. With a highly developed genetic system and expression vectors, *Hfx. volcanii* seems to be ideally suited for the homologous overproduction of proteins and the heterologous overproduction of proteins of other halophiles with the aim of crystallization and structure determination. 

Although the genetic system of *H. volcanii* is highly developed, a system for effective transposon mutagenesis is still missing. An early attempt to construct transposons for haloarchaea did not lead to the desired result [129]; therefore, *in vivo* transposon mutagenesis might be difficult to achieve in haloarchaea with a high cytoplasmic salt concentration. However, *in vitro* transposon mutagenesis of a genomic library followed by transformation seems to be feasible and has been successfully established for genetically more “complicated” species, such as *Streptomyces* [91]. Similarly, there seems to be no technical obstacle against using a library with genomic fragments well below the average gene size in a selectable vector to perform genome-wide random insertional mutagenesis.

As outlined above, the methods for high-throughput genetics and phenotyping have been already developed, and a community effort would be needed to make *Hfx. volcanii* the first archaeal species for which a collection of deletion mutants for all genes could be realized. Last but not least, as a halophile that lyses under low salt conditions, *Hfx. volcanii* is a biologically safe species that could be used very generally for teaching purposes in schools and for public scientific awareness.
